# Supplementary material for: Tande nou gwonde! (Hear us roar!)- Youth perspectives of maternal near-misses: Protocol for a photovoice study of young childbearing people’s perspectives of maternal near-misses in northwest Haiti
Source: PLoS One. 2024 May 17;19(5):e0303168. doi: 10.1371/journal.pone.0303168 (PMC11101082; doi:10.1371/journal.pone.0303168)
Supplement: S1 Appendix — (DOCX) [file pone.0303168.s001.docx]

Supporting information

S1 Appendix. Qualitative research design, strategy, and characteristics

We will consider several characteristics of qualitative research for this study: natural setting, participants and researcher(s) as key instrument, project transparency and multiple sources of data, participants’ meanings, reflexivity, and holistic account [1].

Natural setting

We will reach our participants in their natural environment and context through various safe and secure means to undertake face-to-face interactions within CMB’s catchment area within this northwest part of Haiti. Travel to Haiti of the RA and RL is not feasible currently or in the foreseeable future (due to ongoing political chaos, community unrest and suffering in Haiti, travel restrictions and the COVID-19 pandemic). We have found other ways to proceed and work closely with our Haitian partners, and across borders. First, our south-north CRT members will meet regularly via phone or teleconferencing. Second, at critical points of the project, the RA and RL (both in Canada) will meet in person, and then involve other members by distance. Third, the Photovoice workshop, semi-structured interviews and small group discussions will be facilitated by the LRC and include involvement of the RA and RL via phone and teleconferencing wherever possible and appropriate.

Participants and researchers as key instrument

The diversity of participants and of researchers (our CRT) will contribute in different ways as it relates to community knowledge, skills, availability, and expertise.

- 1. Participants: will be young women/gender diverse childbearing people (age 15-24) from varying areas rural and remote within the catchment area, and with diverse near-miss experiences. They will use mobile phone photography as a research tool. As part of the iGeneration, we understand these youth to be digitally connected and at ease with mobile phone camera use. There is widespread use of mobile phones in Haiti with 63% of population making mobile phone connections [2]. And there is increasing digital growth in mobile phone connections, internet and social media use, especially among Haitian youth [2]. The participants are central to this project; their participation will inform next steps and necessary adaptations in the research process.

- 1. The Local Research Coordinator (LRC): will be a female young person from the community, chosen for their community involvement, interest in research and this project, and ideally, their connection with CMB (e.g., working as nurse or community health worker) and any previous research involvement. This young person’s ability to liaise within the community and interface with CMB staff/administration/Board of Directors will be vital to maximizing youth participation and engagement and CRT involvement. The LRC will work closely with the Local Obstetrician (LO), to coordinate “on the ground” aspects of the study. The LRC will also co-facilitate the Photovoice workshop, co-lead during interviews and group discussions, and assist with data analysis. The LRC will bring a youth and community perspective to the CRT.

- 1. The Local Obstetrician (LO): OD is a long-time member of the community and current Assistant Medical Director of CMB (and co-author of one of the RL’s PhD thesis papers). The LO will advise on local community goals and CMB strategic goals to improve maternal health and well-being, goals of this project, study development and logistics, cultural awareness, recruitment, ethical considerations, budget preparation, and security issues. The LO will bring a clinical and community perspective to the CRT.
  2. The Community Health Lead (CHL): RABA is a member of the community and current lead for CMB’s community health department. The CHL will bring a community and clinical perspective of community health to the CRT and advise/assist in many aspects of project.
  3. The Research Assistant (RA): MCC is a Haitian Nurse-Midwife now living in Canada, and an experienced healthcare professional with community development and research experience in Haiti (and co-author of the RL’s Master’s work). The RA will support study development, cultural awareness, logistics, recruitment, interviews and small group discussions, translation, and ethical considerations. The RA will bring a clinical and cross-cultural perspective to the CRT.
  4. The Research Lead (RL): TM is a Canadian PhD candidate and Registered Midwife with work, volunteer, and research experience in Haiti. The RL will lead all aspects of the study, its development, funding, and ethics approval, with cultural awareness, sensitivity, and humility. The RL will bring a clinical and academic perspective to the CRT.

All members of the CRT as well as consenting participants will be involved in knowledge translation and mobilisation activities, and as co-authors of the final study report.

Project transparency and multiple sources of data

Leaders of the community’s hospital support this research being done. Regarding cultural appropriateness of this research, they emphasize the importance of project transparency within the community at large. They believe that by providing a concise explanation of the study methodology and having clear guidelines regarding ethical conduct within the project (especially as it relates to photography), that this project will be welcomed. We will use personal networks to inform the community about this project and modify it as necessary. We will ensure development of community-specific and appropriate recruitment materials and strategies. We will consider if additional sources of data are needed or available to support this research, e.g., hospital records, participant field notes.

Participants’ meanings

From a feminist lens, we aim to create a safe space for female participants “to cast their voice in a world that often drowns out their perspective” [3: Aboulkacem, p.876]. We go beyond the adage “a picture is worth a thousand words” in our Photovoice project. Giving voice reflects Wang’s original acronym of VOICE: **V**oicing **O**ur **I**ndividual and **C**ollective **E**xperience [4: Wang, p.381]. We will also elicit participant’ meanings as they talk about their photographs, and use their images to communicate their lived experiences, expertise, and knowledge. We will illuminate participant-chosen photographs and amplify the voices of Haitian youth to centre understanding of near-misses among Haitian youth from *their* perspectives and through *their* narratives. Participants’ meanings will also be captured during Photovoice workshops, undertaking member check-in with participants (e.g., regarding their narratives), including key quotes, and involving participants in development of knowledge translation and mobilization strategies and activities.

Reflexivity

First, this study is premised on the RL having built trusted community relationships. The RL approaches this study through a lens of equity, social justice, and sustainable community development. Second, the RL is committed to working as part of a south-north collaboration and partnership, in which the research will span communities, cultures and languages. Third, the RL understands the social justice underpinnings required of a community-based researcher, and the importance of “knowing who I am ” [5: Hall, p.156] and being responsible for the actions taken within the research. As one of the co-researchers, the RL (she/her/elle) positions herself as a white, settler Canadian, middle-aged female who identifies as a woman, a mother, a teacher, a midwife, and a doctoral student. The RL is not a woman of colour, not Haitian, and not confronted with constant worry of maternal near-misses in her community (even as a midwife). The RL recognizes that our research is meant to take place in someone else’s community, a community that she has been invited as a guest, to be a part of over the past several years and who has grown to become a community ally. Previous work and research experiences in Haiti first led the RL to this community of interest that she continues to work with as a midwife educator and PhD student. As one of the co-researchers for this study, the RL acknowledges how she stands to gain academically and professionally from the completion of such a research project.

Holistic account

We aim to create a visual representation of our central phenomenon of near-misses among youth in Haiti. This visual representation will be in addition to photographs included in our account. In a culturally appropriate way, we expect that through the diversity of our CRT and supported by the RL’s PhD advisors, we will be able to develop a visual model of our findings, that can be used for knowledge mobilization.

References

1. Creswell J. Research design: qualitative, quantitative, and mixed methods approaches-4th edition. Thousand Oaks, California: SAGE Publications; 2014.

2. DataReportal: Digital 2020: Haiti. <https://datareportal.com/reports/digital-2020-haiti> (2021). Accessed 2021 14 January.

3. Aboulkacem AR, Aboulkacem S, Haas LE. Photovoice 2.0: a comprehensive research framework for the digital generation. TechTrends. 2021;65(5):874-83.

4. Wang C, Burris MA. Photovoice: concept, methodology, and use for participatory needs assessment. Health Educ Behav. 1997;24(3):369-87.

5. Hall BL. Learning to listen: foundations of teaching and facilitating participatory and community-based research In: Etmanski C, Hall BL, Dawson T, editors. Learning and teaching community-based research : linking pedagogy to practice. Toronto: University of Toronto Press; 2014. p.162.
