# Supplementary material for: Tande nou gwonde! (Hear us roar!)- Youth perspectives of maternal near-misses: Protocol for a photovoice study of young childbearing people’s perspectives of maternal near-misses in northwest Haiti
Source: PLoS One. 2024 May 17;19(5):e0303168. doi: 10.1371/journal.pone.0303168 (PMC11101082; doi:10.1371/journal.pone.0303168)
Supplement: S1 File — (DOCX) [file pone.0303168.s002.docx]

Supporting information

S1 File. Data collection forms, interview guide, and participant pre-workshop and end-of-project questionnaires (also available in Creole and French).

| Item | Description of item | Page |
| --- | --- | --- |
| 1. | Data Collection Form for Individual Interviews | 2 |
| 2. | Data Collection Form for Photos | 4 |
| 3. | Individual Interview Guide | 5 |
| 4. | “SHOWED” Focus Group Discussion Guide | 6 |
| 5. | “SHOWED” Data Collection Sheet | 7 |
| 6. | Participant pre-workshop questionnaire | 8 |
| 7. | Participant end-of-project questionnaire | 10 |

**Project Hear Us Roar!**

**Data Collection Form for Individual Interviews**

**Data Collection Form for Interviews:**

| **Participant number** | **Chosen name or pseudonym** | **Consent**  **obtained/**  **copy provided** | **Interviewer initials/**  **date/time** | **Interview location** | **Observations/**  **notes or comments** | **Length**  **of interview** | **Compensation provided/**  **interviewer**  **initials** | **e-File name** |
| --- | --- | --- | --- | --- | --- | --- | --- | --- |
| P1 |  |  |  |  |  |  |  |  |
| P2 |  |  |  |  |  |  |  |  |
| P3 |  |  |  |  |  |  |  |  |
| P4 |  |  |  |  |  |  |  |  |
| P5 |  |  |  |  |  |  |  |  |
| P6 |  |  |  |  |  |  |  |  |
| P7 |  |  |  |  |  |  |  |  |
| P8 |  |  |  |  |  |  |  |  |
| P9 |  |  |  |  |  |  |  |  |
| P10 |  |  |  |  |  |  |  |  |
| P11 |  |  |  |  |  |  |  |  |
| P12 |  |  |  |  |  |  |  |  |
| P13 |  |  |  |  |  |  |  |  |
| P14 |  |  |  |  |  |  |  |  |
| P15 |  |  |  |  |  |  |  |  |

**Personal Information:**

| **Participant number** | **Sex/Gender/**  **Pronouns**  (e.g., female, woman, they) | **Age** | **Marital status** (single, in relationship, married, in common law relationship, separated, divorced) | **Living situation**  (on own, w/ family, w/friends, w/partner, other) | **Living location** (urban, semi-urban, rural, other) | **Education**  (grade school, high school, technical training, some college/university, college/university diploma) | **Age at time of MNM** | **Location of MNM**  (at home, en route to HCF, at a HCF, at CMB) |
| --- | --- | --- | --- | --- | --- | --- | --- | --- |
| P1 |  |  |  |  |  |  |  |  |
| P2 |  |  |  |  |  |  |  |  |
| P3 |  |  |  |  |  |  |  |  |
| P4 |  |  |  |  |  |  |  |  |
| P5 |  |  |  |  |  |  |  |  |
| P6 |  |  |  |  |  |  |  |  |
| P7 |  |  |  |  |  |  |  |  |
| P8 |  |  |  |  |  |  |  |  |
| P9 |  |  |  |  |  |  |  |  |
| P10 |  |  |  |  |  |  |  |  |
| P11 |  |  |  |  |  |  |  |  |
| P12 |  |  |  |  |  |  |  |  |
| P13 |  |  |  |  |  |  |  |  |
| P14 |  |  |  |  |  |  |  |  |
| P15 |  |  |  |  |  |  |  |  |

**Abbreviations:** w/=with, HCF=healthcare facility, CMB=Centre Médical Béraca

**Project Hear Us Roar!**

**Data Collection Form for Photos**

**Data Collection Form for Photos:**

| **Participant numberPhoto#** | **Chosen name or pseudonym**  **for photo** | **Ongoing consent**  **obtained** | **Short description of photo** | **Title or caption of photo** | **Copy requested** | **Copy provided** | **e-File name** |
| --- | --- | --- | --- | --- | --- | --- | --- |
| P#ph# |  |  |  |  |  |  |  |
|  |  |  |  |  |  |  |  |
|  |  |  |  |  |  |  |  |
|  |  |  |  |  |  |  |  |
|  |  |  |  |  |  |  |  |
|  |  |  |  |  |  |  |  |
|  |  |  |  |  |  |  |  |
|  |  |  |  |  |  |  |  |

**Project Hear Us Roar!**

**Individual Interview Guide**

*to be used by Local Research Coordinator as a guide to semi-structured individual interviews with participants after they have completed their photography in the community

**Date: ___________________**

**Form being completed by: ________________________**

**Participant ID or Pseudonym**: ______________________________

*We would like to know how your experience was of taking photos in the community for Project Hear Us Roar. Today, I am looking forward to hearing about that experience, seeing your photos and listening to your stories. By the end of the interview, I will need you to make a selection of the photos that are most important or meaningful to you.*

**Questions**

1. How did you find your experience of taking photos?
2. When you look back at all your photos, what comes to your mind?
3. How would describe what you took photos of?
4. How does your lived experience of surviving a maternal near-miss (MNM) relate to these photos?
5. Could you pick out a few photos that really stand out for you? LRC referring to each particular photo participant has picked out, asks: How does this picture make you feel? What message is the photo trying to convey?
6. Of all the photos you took, would you please make a selection of the most important or meaningful ones? These are the photos that we will then bring and share at our small focus group discussion with the other participants (who will also bring and share their most important photos too).

***Thank you for your participation!***

**Project Hear Us Roar!**

**“SHOWED” Focus Group Discussion Guide**

*to be used by Local Research Coordinator as a guide to small focus group discussions (participants from Group A and from Group B) and photo elicitation

**Date: ___________________**

**Form being completed by: ________________________**

**Name of participant**: ______________________________

*As a group, we would like to know more about your experiences of taking photos in the community for Project Hear Us Roar. Today, I am looking forward to hearing about those experiences, seeing your most meaningful photos and listening to your important stories. Together we will all look at your photos and talk about what we think they mean as a group. By the end of our discussion, we will need to decide how we want to display these photos for others to learn about this project, and to hear your voices.*

**Questions**

1. How did you find your experience of taking photos?
2. When you look back at all your photos, what is a general overview of them?
3. How does your lived experience of surviving a maternal near-miss (MNM) relate to these photos?
4. Let’s go through the photos that you each picked out as your most meaningful or important ones. Together we will go through one after the other and answer these questions:

**S**- What do you **S**ee here? (describe what your eyes see)

**H**- What is really **H**appening here? (tell unseen story behind the image)

**O**- How does this relate to **O**ur lives? (explain about how is related to MNMs)

**W- W**hy does this situation, concern, or strength exist? (tell why it is this way)

**E-** How can we become **E**mpowered through our new understanding? (tell how this empowered you or we could educate others about it)

**D-** What can we **D**o about it? (give an action or solution to this)

1. Of all the photos we have, what are your ideas or thoughts about how we could display them for the community to see and for them to hear your voices?

***Thank you for your participation!***

**Project Hear Us Roar!**

**“SHOWED” Data Collection Sheet**

*to be used by Local Research Coordinator during photo elicitation at small focus group discussions

**Date/time/location: ________________________________________________________**

**Form being completed by: ________________________**

**Name of participant**: ______________________________

**Description of photo:** _____________________________

**Title of photo:** ______________________________

**# assigned to photo:** ________________

| **S** | What do you **S**ee here? (describe what your eyes see) |
| --- | --- |
| **H** | What is really **H**appening here? (tell unseen story behind the image) |
| **O** | How does this relate to **O**ur lives? (explain about how is related to MNMs) |
| **W** | **W**hy does this situation, concern, or strength exist? (tell why it is this way) |
| **E** | How can we become **E**mpowered through our new understanding?  (tell how this empowered you or we could educate others about it) |
| **D** | What can we **D**o about it? (give an action or solution to this) |

**Project Hear Us Roar!**

**Participant pre-workshop questionnaire**

*to be read as a script by Local Research Coordinator, or provided to participant to complete after recruitment but prior to Photovoice Workshop

**Date: ___________________**

**Form being completed by: ________________________**

**Participant ID or Pseudonym**: ______________________________

*We would like to know more about you, where you are coming from, and how you are feeling at the start of this photovoice project. This short questionnaire asks some personal questions. Please answer as honestly as possible so we can learn a bit more about who you are and who is participating in this project.*

**Personal background**

Are you female? ________

What pronouns do you use, or how do you identify your gender? ___________

What is your age? _____

What is your relationship or marital status?

___ single

___ in a relationship; not married

___ married

___ in a common law relationship

___ separated or divorced

Where do you live?

___ on your own

___ with family

___ with friend(s)

___ with partner/husband

___ other (please describe)

Where is your place?

___ in city (urban)

___ near city (semi-urban)

___ in countryside (rural)

___ other (please describe)

What is your highest level of education?

___ grade school (up to grade 8)

___ high school (up to grade 12)

___ technical training certificate

___ some college or university training

___ college or university diploma/degree

At what age did you experience your maternal near-miss incident? ____

Where did your maternal near-miss happen?

___ at home

___ en route to a healthcare facility

___ at a healthcare facility

___ at Centre Médical Béraca

**Photovoice background**

How or where did you hear about this photovoice project?

How much experience do you have with taking photos?

___ none

___ very little

___ some

___ quite a bit

___ a lot

Do you own a cellphone that can take photos?

___ yes

___ no, my cellphone can’t take photos

___ no, but I have a digital camera

Do you expect to live in this same area for the next 3 months (to be able to be part of the photovoice project?)

What do you hope to gain from being part of this photovoice project?

What are any questions, worries or concerns you have about being part of this photovoice project?

Other comments:

***Thank you for your participation!***

Adapted from [1]:

1. Dahan R, Dick R, Moll S, Salwach E, Sherman D, Vengris J, et al. Photovoice Hamilton: manual and resource kit. Hamilton ON; 2007.

**Project Hear Us Roar!**

**Participant end-of-project questionnaire**

*to be read as a script by Local Research Coordinator (or provided to participant to complete) at the end the project (i.e., after data collection, analysis, and photo elicitation)

**Date: ___________________**

**Form being completed by: ________________________**

**Participant ID or Pseudonym**: ______________________________

*We are so thankful for all your hard work and dedication throughout this photovoice project. We would like to know from you how it was to be a part of this photovoice project! Please answer as honestly as possible so we can learn and make another project like this better.*

**Photovoice feedback**

How would you rate your overall experience in this photovoice project?

___ Poor

___ OK

___ Good

___ Very good

___ Excellent

How would you rate your enjoyment of the following parts of this photovoice project?

Poor Excellent

Learning about the project at the workshop 1 2 3 4 5

Using the camera to take photos 1 2 3 4 5

Thinking about the message in the photos 1 2 3 4 5

Discussing the photos in an interview 1 2 3 4 5

Writing down your ideas about your photos 1 2 3 4 5

Discussing photos as a small group 1 2 3 4 5

Poor Excellent

Receiving support from others about ideas 1 2 3 4 5

Planning a display/final product 1 2 3 4 5

Communicating ideas to the community 1 2 3 4 5

What was the ***best*** thing for you as part of this photovoice project?

What did you ***find difficult or dislike*** about this photovoice project?

What could we ***change*** to make such a photovoice project better?

Would you recommend photovoice to others?

___ Yes

___ No

___ Maybe (please explain) ___________________________________________________

Other comments:

***Thank you for your participation!***

Adapted from [1]:

1. Dahan R, Dick R, Moll S, Salwach E, Sherman D, Vengris J, et al. Photovoice Hamilton: manual and resource kit. Hamilton ON; 2007.
