# Supplementary figures and images for: Tande nou gwonde! (Hear us roar!)- Youth perspectives of maternal near-misses: Protocol for a photovoice study of young childbearing people’s perspectives of maternal near-misses in northwest Haiti
Source: PLoS One. 2024 May 17;19(5):e0303168. doi: 10.1371/journal.pone.0303168 (PMC11101082; doi:10.1371/journal.pone.0303168)

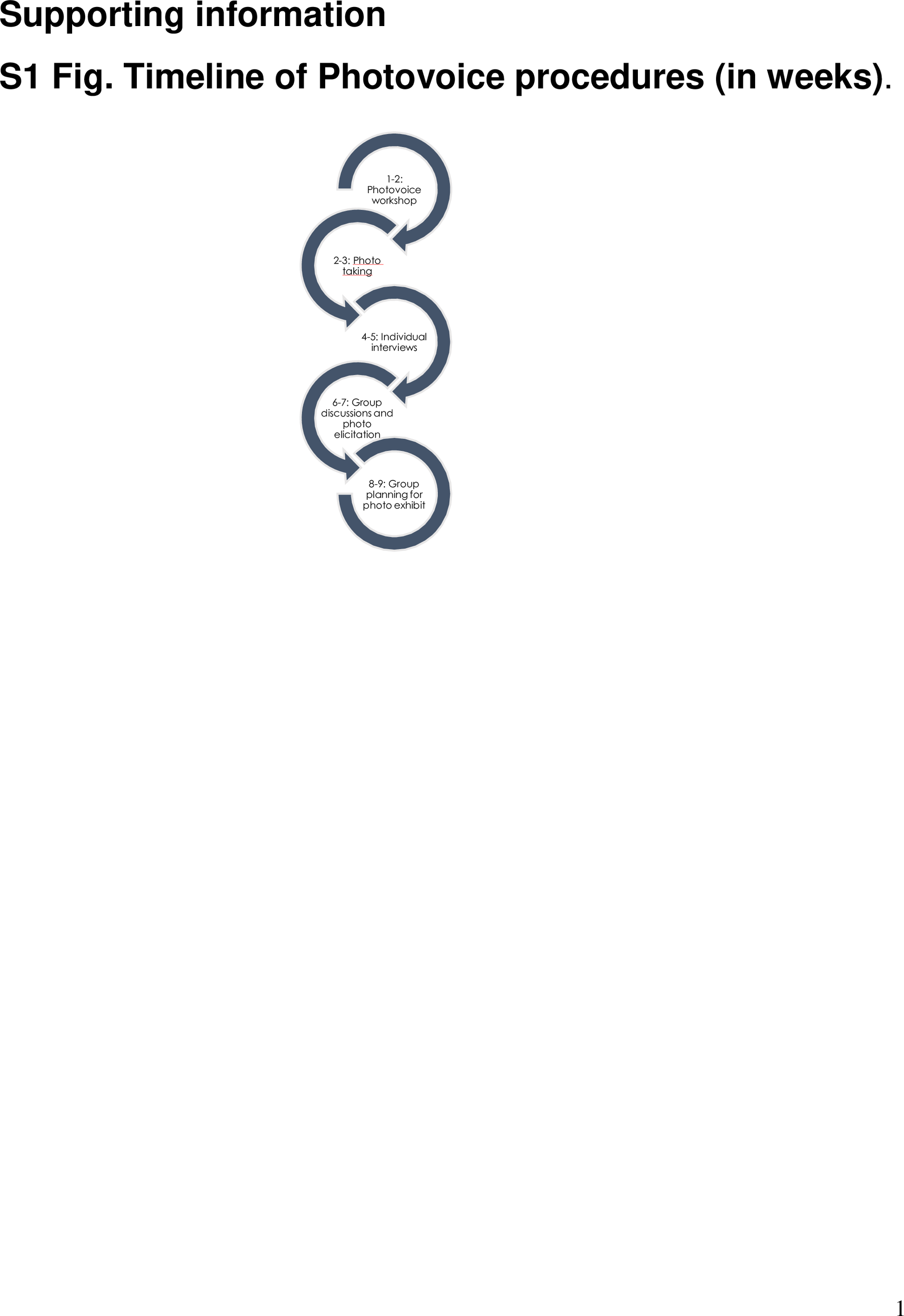

Supplement: S1 Fig — (TIF) [file pone.0303168.s003.tif]
