# Supplementary material for: Tande nou gwonde! (Hear us roar!)- Youth perspectives of maternal near-misses: Protocol for a photovoice study of young childbearing people’s perspectives of maternal near-misses in northwest Haiti
Source: PLoS One. 2024 May 17;19(5):e0303168. doi: 10.1371/journal.pone.0303168 (PMC11101082; doi:10.1371/journal.pone.0303168)
Supplement: Attachment — Submitted filename: Response to reviewers.docx [file pone.0303168.s004.docx]

**Response to reviewers of PLOS ONE**

| **ARTICLE NUMBER:** | PONE-D-23-25467 |
| --- | --- |
| **ARTICLE TITLE:** | Tande nou gwonde! (Hear us roar!)- Youth perspectives of maternal near-misses: protocol for a photovoice study of young childbearing people’s perspectives of maternal near-misses in northwest Haiti |

March 22, 2024

Dear academic editor and reviewers,

Thank you for taking the time to review and make suggestions for a better-quality manuscript. We appreciate your questions, insights, and suggestions that will strengthen our protocol manuscript and ultimately, our study. Please accept this rebuttal letter as a point-by-point response to your review. Note: line numbers in this letter correspond to the line numbers in the ‘Revised Manuscript with Track Changes.docx’.

**JOURNAL Requirements and responses:**

1. Please ensure that your manuscript meets PLOS ONE's style requirements, including those for file naming.

**Response:**

Thank you for this reminder. We have consulted guidelines for style requirements and file naming and made changes as necessary.

2. Please include your full ethics statement in the ‘Methods’ section of your manuscript file. In your statement, please include the full name of the IRB or ethics committee who approved or waived your study, as well as whether or not you obtained informed written or verbal consent. If consent was waived for your study, please include this information in your statement as well.

**Response:**

We have moved our full ethics statement from the end of our protocol to the Methods section of our manuscript and added the component about obtaining consent. We have also made other additions based on responses to Reviewer 1. Note that is a protocol for a planned study and therefore consent has not been obtained yet. The full ethics statement, found on Lines 234-265 now reads:

**“Ethics**

This research will have human participants, use photographs of their community, and will also use Personal Health Information (PHI) to identify potential participants. Our study will be guided by Tri-Council Policy Statement (TCPS): Ethical Conduct For Research Involving Humans [1]. Ethics approval has been received in writing from the Research Ethics Committee of Centre Médical Béraca, Haiti’s Northwest Department of Health and the Hamilton Integrated Research Ethics Board (HiREB) [2] (project ID#: 14954) in Hamilton ON, Canada. Only participants who provide their written or marked, informed, and on-going consent to participate will be included in the study.

Our study will integrate WHO-recommended guidelines for the ethical and safe conduct of intervention research on domestic violence against women [3]. In addition, we will take a trauma- and violence-informed approach to this research, using a framework that directs particular ethical attention to participant safety [4]. We understand the potential for photographic images to be intrusive and to lead to unintended consequences. We will refer to guidelines that address ethical issues raised by the use of photovoice and ensure we exceed Wang and Redwood-Jones’ minimum best practices of photovoice ethics [5].

Research participants will keep all their own digital photos taken during fieldwork. Photos they wish to share for the study (including for public display) will be transferred electronically, deidentified and saved with a unique study identifier or code number. To protect the privacy of those who might appear in photos (or their property), we will blur identifying aspects of photos. Transcripts of interviews and focus group discussions will be de-identified during transcription. Only designated person will be allowed to access the study key file and assign unique study identifiers or codes to participants. The study key file will be securely stored on MacDrive, a privately hosted, secure, cloud storage at McMaster University, Hamilton ON, Canada. Computer tablets for audio-visual files will be housed in a locked secure location in the CMB office of the LO. At the end of the project, the community will have possession of the new knowledge (all the de-identified data) generated by the community. CMB will securely store these data. McMaster University will also have a copy of these data on the secure MacDrive. We will permanently delete all transcripts and translations five years after a community presentation or research paper publication. A digital copy of photos will be kept by the research team on the secure MacDrive when they are not displayed, and until five years after a community presentation or research paper publication, at which time they will be destroyed.”

3. We note that the original protocol that you have uploaded as a Supporting Information file contains an institutional logo. As this logo is likely copyrighted, we ask that you please remove it from this file and upload an updated version upon resubmission.

**Response:**

We do not notice any logos in our Supplementary files. If you referring to S1_Fig, there is an inserted “SmartArt text” process icon provided as part of Microsoft Word. We have left this figure as is since we do not believe that it is copyrighted.

4. Please review your reference list to ensure that it is complete and correct. If you have cited papers that have been retracted, please include the rationale for doing so in the manuscript text or remove these references and replace them with relevant current references. Any changes to the reference list should be mentioned in the rebuttal letter that accompanies your revised manuscript. If you need to cite a retracted article, indicate the article’s retracted status in the References list and also include a citation and full reference for the retraction notice.

**Response:**

We are not aware of any cited papers in our manuscript which have been retracted. However, we have made a few additions to our reference list. These additional references are (see these listed at the end of this letter, and integrated into the References in the actual manuscript):

- DataReportal [6]
- NVivo [7]
- Institut Haïtien de l’Enfance (IHE), et ICF [8]
- Papers by: Jean-Baptiste M, Millien C, Pognon PR, Casella Jean-Baptiste M [9]; and by Obrillant D [10].

**REVIEWER 1: comments and responses**

This will be an interesting study if conducted, however, I have a few comments which if the authors address will help strengthen the protocol and the future study.

**Response:**

Thank you for the recognition of this study’s potential. We also appreciate the comments you have provided us that will no doubt strengthen our work.

1. **Introduction:**
a) In line 81, the authors should capitalize each word in the low-middle income country to read Low-Middle Income Country (LMIC).

**Response:**

Thank you for this suggestion. We have made this change on Line 93.

b) In line 82, the reference [5] should come before the full stop. The same change should be made in 121 for reference [18].

**Response:**

We appreciate your keen eye to this oversight. We have made this change on Lines 94 and 140.

2. **Photovoice methodology:**

The description of the process of the photovoice, how many photographs will each participant take at a point?

**Response:**

Thank you for your question as this led us to change the wording about the process of Photovoice. Please see the changes made on Line 329-331. The sentence now reads: “This process will have participants take their own photos using **a cell phone** and **later they will be asked to** make choices about which photos most effectively represent their near-miss experiences.”

We also have made some changes in Lines 331-333 to answer your question and better tie these sentences together. The sentence now reads: “No instructions will be given regarding who, what, when, where, or how many photographs to take **during their photo taking fieldwork** [11], creating the opportunity for participants to be empowered to “find their own voice”.

3. **Data collection:**
a) What is the operational definition for maternal near-misses (MNMs) in this particular study? What variable are authors looking at in defining this maternal near-misses?

**Response:**

We appreciate these questions. They point to our need to clarify and better explain the operationalization of MNMs in the manuscript. The operational definitions and variables for a MNM in our study are now better described by the addition of this paragraph to the manuscript. Please see Lines 277-286: “To identify cases of MNMs that occurred in a healthcare facility (Group A), we will first search hospital records for maternal cases that included conditions or complications such as severe postpartum hemorrhage, severe pre-eclampsia, eclampsia, sepsis or severe systemic infection, and ruptured uterus. MNM criteria would include clinical organ dysfunction (e.g., shock, uncontrollable fit, stroke), laboratory markers of organ dysfunction, or clinical management proxies (e.g., hysterectomy, blood transfusion, dialysis). To identify cases of MNMs that occurred at home (Group B), we will seek out participants who self-report having had a very grave obstetric incident during pregnancy, labour or childbirth, or the six weeks after the termination of their pregnancy, such that from their perspective, they narrowly escaped death due to some kind of intervention or help, outside of the healthcare system.”

b) Will there be a review of maternal records from the health facilities? If yes. What about the participant that have not attended a health facility but will be involved in this study?

**Response:**

Yes, we will undertake a review of maternal records from the healthcare facility of Centre Médical Bérara (CMB). With our MNM criteria in mind, this will be done with input from nursing personnel, and led by our community research team (CRT) member, Dr. Olès Dorcely, who is an Obstetrician-Gynecologist (please see Lines 300-301 which already describe this). To provide more clarity, though, we have made an addition found on Lines 301-303 that reads: “Together with other members of the CRT, LO will confirm eligibility such that the sample of cases meets our operational definition of MNMs.”

In the manuscript, please also see Lines 282-286 regarding participants whose MNM occurred at home (Group B) and their involvement in the study.

4. **Ethical issues:**
a) How will the photographs and other data collected by the participants be handled, stored, and managed to ensure the confidentiality and privacy of the participants themselves and those that might appear in the photographs?

**Response:**

Thank you for your important attention to specifics about ethics.

About data handling, storage, privacy, and confidentiality, to Lines 252-260 we have added: “Research participants will keep all their own digital photos taken during fieldwork. Photos they wish to share for the study (including for public display) will be transferred electronically, deidentified and saved with a unique study identifier or code number. To protect the privacy of those who might appear in photos (or their property), we will blur identifying aspects of photos. Transcripts of interviews and focus group discussions will be de-identified during transcription. Only designated person will be allowed to access the study key file and assign unique study identifiers or codes to participants. The study key file will be securely stored on MacDrive, a privately hosted, secure, cloud storage at McMaster University, Hamilton ON Canada. Tablets for audio-visual files will be password-protected and housed in a locked secure location in the CMB office of the LO.”

b) And what will happen to those photographs taken after the study?

**Response:**

Regarding data retention, to Lines 260-265 we have added: “At the end of the project, the community will have possession of the new knowledge (all the de-identified data) generated by the community. CMB will securely store these data. McMaster University will also have a copy of these data on the secure MacDrive. We will permanently delete all transcripts and translations five years after a community presentation or research paper publication. A digital copy of photos will be kept by the research team on the secure MacDrive when they are not displayed, and until five years after a community presentation or research paper publication, at which time they will be destroyed.”

c) How will the consenting of the participants who could be under 18 years old be taken? Or the authors do not anticipate that? This is a case where the study is among young women.

**Response:**

Your questions about informed consent and age are appreciated. In accordance with the Hamilton Integrated Research Ethics Board (HiREB), the age of majority is considered age 16+ years. Individuals under the age of 16 years are considered minors. For clarity around this concern, we have added the following to Lines 363-369: “In many jurisdictions (including the Province of Ontario, Canada), capacity for providing consent is not based on age, but on competency. In Haiti the capacity to provide consent is likewise not based on age but on the person’s competency. A consent approach based on competency for those under age 16, adheres to local regulations in this Haitian community. Thus, local researchers will confirm participant competency (i.e., individual’s ability to understand what study participation means and ability to decide on study participation) and proceed with consent by capacity for participants who are age 15; they will not obtain parental consent.”

5. **Analysis:**

How will the analysis of the narrative of transcripts be done? Will there be the use of any qualitative analysis software? If there is one, which one will be used?

**Response:**

We neglected to include information about how analysis of the narrative transcripts will be done. Thank you for pointing out this oversight. We have added this in response, to Lines 425-426:

“To assist with data handling throughout analysis, we will upload narrative data into NVivo^TM^ (version 14), a software for qualitative data analysis.”

**REVIEWER 2: comments and responses**

This is a very well written research protocol on an important and understudied topic. The authors make a clear justification for why the collection of qualitative data about maternal near-misses among youth in Haiti is warranted (i.e.: survivors of maternal near-misses act as a ‘proxy’ group). Definitions of youth/adolescence are well described as is the explanation for including gender diverse childbearing people in recruitment. Excellent justification for the use of Photovoice methodology and CBPR framework. Good inclusion of a positionality statement. The authors provided a very detailed and thoughtful explanation about the empowering and emancipatory potential of this research study. Below are some minor comments/suggestions:

**Response:** Thank you very much for your comments. They are much appreciated.

1. **Introduction:**

Lines 85-86: The sentence: “One of the four leading causes of death for those 20-24 years old is pregnancy, childbirth and the weeks after birth or at the end of a pregnancy” is awkward. Could it be re-worded to say: “One of the four leading causes of death for those 20-24 years old is pregnancy, childbirth and the weeks after birth”.

**Response:**

We welcome your suggestion of rewording this awkward sentence. We have simplified the wording in Lines 102: “One of the four leading causes of death for those 20-24 years old is pregnancy-related.”

2. **Sampling:**

Lines 233-234: Is there any information that can be included as to whether more births in Haiti occur in healthcare facilities or in the community/at home?

**Response:**

We have added the following sentence to the Introduction section , to provide additional context. This is found in Lines 95-100: “According to Haitian national data from 2018, forty-two percent (42%) of births were attended by skilled birth personnel, thirty-nine percent (39%) of births were attended at health facilities, and about sixty-one percent (61%) of births took place in the home setting [8]. Haitian Traditional Birth Attendants (TBAs), called ‘Matwon’, support about fifty percent (50%) of the births in Haiti [8]. Matwon attend to labouring individuals in the home setting, often in remotes areas that lack adequate medical and material resources [9, 10].

3. **Recruitment:**

Access to a cell phone should be listed as an inclusion criterion to participate. Is it assumed that the majority of youth (of all socioeconomic statuses) in Haiti have access to cell phones with adequate storage for photos and access to data plans and chargers for their phones?

**Response:**

We appreciate these questions about digital access and equity in Haiti among youth. According to data about how people in Haiti use digital devices and services in 2024, it is estimated that 82.7% of the total population of Haiti makes cellular mobile connections [6]. Unfortunately, disaggregated data by age and socioeconomic status were not located. Our Haitian team members remind us that it is very common for youth to have a mobile device, they purchase data on an as-need basis, and they will often make use of solar chargers for their phones. If a participant has access to cell phone, we will provide some compensation to purchase data (describe in 4. Compensation). We responded to this concern on Lines 313-314 by adding: “To be included in the study, participants will need a cell phone with built-in camera or have access to one for temporary use.”

4. **Compensation:**
a) Line 319: Nice explanation. Is the total maximum monetary compensation USD$30? (workshop, photo taking/interview and focus group discussion)?

**Response:**

Thank you for kind words and for pointing out that the total maximum monetary compensation is $30 USD. To make this clearer in the text we have used your words. On Lines 390-392 it now reads: “Compensation will be: $10 USD/participant for each “activity”: workshop, photo taking/interview and focus group discussion (**i.e., a total maximum monetary compensation of $30 USD**).”

b) Please provide some context about the small gift of cellphone minutes.

**Response:**

In terms of providing context about cellphone minutes, we have further explained in Lines 393-394 and it now reads: “Participants also be offered healthy snacks during workshop sessions, interviews and group discussions, a small gift of healthcare supplies, and **a specific amount of data usage or calling minutes purchased in advance (for data and to access the CRT as needed)**.”

**References**

1.	Canadian Institutes of Health Research (CIHR), Natural Sciences and Engineering Research Council of Canada (NSERC), Social Sciences and Humanities Research Council (SSHRC). Tri-council policy statement: Ethical conduct for research involving humans. Ottawa: Secretariat on Responsible Conduct of Research; 2018.

2.	Hamilton Integrated Research Ethics Board (HiREB): <https://hireb.ca/> (2018). Accessed 2021 8 September.

3.	World Health Organization (WHO). Ethical and safety recommendations for intervention research on violence against women: Building on lessons from the WHO publication “Putting women first: ethical and safety recommendations for research on domestic violence against women”. Geneva: WHO; 2016.

4.	Nonomura R, Giesbrecht C, Jivraj T, Lapp A, Bax K, Jenney A, et al. Toward a trauma- and violence-informed research ethics module: considerations and recommendations. London, Ontario: Western University; 2020.

5.	Wang CC, Redwood-Jones YA. Photovoice ethics: perspectives from Flint Photovoice. Health Educ Behav. 2001;28(5):560-72.

6.	DataReportal: Digital 2024: Haiti. <https://datareportal.com/reports/digital-2024-haiti> (2024). Accessed 2024 11 March.

7.	NVivo (Version 14) [Computer software], Luminero, www.lumivero.com. 2023.

8.	Institut Haïtien de l’Enfance (IHE), et ICF. Enquête mortalité, morbidité et utilisation des services (EMMUS-VI 2016-2017). Pétion-Ville, Haïti, et Rockville, Maryland, USA.: IHE et ICF; 2018.

9.	Jean-Baptiste M, Millien C, Pognon PR, Casella Jean-Baptiste M. Reframing the three delays framework: factors influencing referrals to facilities by matrones in rural Haiti. BMJ Glob Health. 2023;8(11).

10.	Obrillant D. « Le rôle des matrones dans la gestion et l’utilisation durable de la biodiversité en Haïti. Matrimonialisation et sauvegarde de leur métier » The role of matrons in the conservation and sustainable use of biodiversity in Haiti. Matrimonialization and safeguarding of their profession. Etudes caribéennes [en ligne] [Internet]. 2020 2024 11 March; 45-46(Avril-Août 2020). Available from: <http://journals.openedition.org/etudescaribeennes/18967>

11.	Daniel C, Logie C. Contexts of risk: a photo-voice study of Haitian youth perceptions of their HIV risk. Glob Soc Welf. 2016;3(4):255-67.
