# Supplementary material for: Tande nou gwonde! (Hear us roar!)- Youth perspectives of maternal near-misses: Protocol for a photovoice study of young childbearing people’s perspectives of maternal near-misses in northwest Haiti
Source: PLoS One. 2024 May 17;19(5):e0303168. doi: 10.1371/journal.pone.0303168 (PMC11101082; doi:10.1371/journal.pone.0303168)
Supplement: Attachment — Submitted filename: Response to Reviewers.docx [file pone.0303168.s005.docx]

**Response to reviewers of PLOS ONE**

| **ARTICLE NUMBER:** | PONE-D-23-25467 |
| --- | --- |
| **ARTICLE TITLE:** | Tande nou gwonde! (Hear us roar!)- Youth perspectives of maternal near-misses: protocol for a photovoice study of young childbearing people’s perspectives of maternal near-misses in northwest Haiti |

April 12, 2024

Dear academic editor and reviewer #1, Dr. Robert Kokou Dowou,

Thank you for taking the time once again to review with great attention our manuscript. We appreciate your two further questions and suggestions meant to strengthen our protocol manuscript and ultimately, our study. Please accept this rebuttal letter as a point-by-point response to the points you raised. Note: line numbers in this letter correspond to the line numbers in the ‘Revised Manuscript with Track Changes.docx’.

**REVIEWER 1: comments and responses**

I commend you on your efforts to address all the previous comments, however, I still have a few clarifications and suggestions. I pray you can accept this in good faith and address them**.**

**Response:**

Thank you for your support of this work. We are happy to address your comments!

1. **Introduction:**
a) In Line 98: Are the Haitian Traditional Birth Attendants (TBAs) an officially recognised part of the Haitian health system? Are they regulated? If yes, it should be indicated, or otherwise, a statement of clarification should be indicated of their clandestine and unauthorized nature.

**Response:**

Haitian Traditional Birth Attendants called ‘matwon’ or ‘fanm chay’ are not officially recognised as part of the health system in Haiti. We have added wording ‘fanm chay” and specified the unrecognised but valued support of these Haitian TBAs. On Lines 98-103 this now reads as:

“Haitian Traditional Birth Attendants (TBAs), called ‘matwon’ or ‘fanm chay’, support about fifty percent (50%) of the births in Haiti [1]. Currently, these TBAs are not supported by Haiti’s public health ministry which exclusively advocates for “skilled birth attendance” in health facilities [2, 3]. Nonetheless, as valued, trusted, and knowledgeable birth attendants, ‘matwon’ support labouring individuals in the home setting, often in remotes areas that lack adequate medical and material resources [4-6].”

2. **Ethics:**
b) In Line 296: The full meaning of LO should be provided when being used for the first time before the subsequent use of the abbreviation.

**Response:**

Thank you for finding this oversight. We note that LO actually appears earlier in the manuscript, and it was not defined when first used. We have made the change and clarified that the Local Obstetrician is also part of the Community Research Team. These changes are found on Lines 253-254 and read as: “Computer tablets for audio-visual files will be housed in a locked secure location in the CMB office of the Local Obstetrician (LO), a member of the community research team.”

3. **Obtaining informed consent:**

Ethics: I am still not satisfied by the justification given on the consent/parental ascent for participants that are below 16 years. You mention that "In Haiti the capacity to provide consent is likewise not based on age but on the person’s competency”. It will be very helpful if the authors can provide any reference to this statement.

**Response:**

Thanks for the suggestion to provide a reference about our statement on consent and competency. We have provided a reference from Haiti’s national legislation regarding age of consent being 15-years old and changed the wording of these sentences. On Lines 358-360, it now reads: “In Haiti, according to national child protection legislation the age of consent is 15-years old [7]. A consent approach based on competency for those under age 16, adheres to national legislation and aligns with local expectations in this Haitian community.”

**JOURNAL requirements and responses:**

1. Please review your reference list to ensure that it is complete and correct. Any changes to the reference list should be mentioned in the rebuttal letter that accompanies your revised manuscript.

**Response:**

We have made a few additions to our reference list. These additional references are (see these listed at the end of this letter, and integrated into the References in the actual manuscript):

Papers by:

- Dev, Kivland, Faustin, Turnier, Bell and Leger
- Ministère de la Santê Publique et de la Population (Haïti) (MSPP)
- Raymondville et al.
- International Centre for Missing & Exploited Children (ICME)

**References**

1.	Institut Haïtien de l’Enfance (IHE), et ICF. Enquête mortalité, morbidité et utilisation des services (EMMUS-VI 2016-2017). Pétion-Ville, Haïti, et Rockville, Maryland, USA.: IHE et ICF; 2018.

2.	Dev A, Kivland C, Faustin M, Turnier O, Bell T, Leger MD. Perceptions of isolation during facility births in Haiti - a qualitative study. Reprod Health. 2019;16(1):185.

3.	Ministère de la Santê Publique et de la Population (Haïti) (MSPP). Plan stratégique national de santé sexuelle et reproductive 2019-2023/strategic plan for sexual and reproductive health (2019-2023). Port-au-Prince, Haiti: MSPP; 2019.

4.	Jean-Baptiste M, Millien C, Pognon PR, Casella Jean-Baptiste M. Reframing the three delays framework: factors influencing referrals to facilities by matrones in rural Haiti. BMJ Glob Health. 2023;8(11).

5.	Obrillant D. « Le rôle des matrones dans la gestion et l’utilisation durable de la biodiversité en Haïti. Matrimonialisation et sauvegarde de leur métier » The role of matrons in the conservation and sustainable use of biodiversity in Haiti. Matrimonialization and safeguarding of their profession. Etudes caribéennes [en ligne] [Internet]. 2020 2024 11 March; 45-46(Avril-Août 2020). Available from: <http://journals.openedition.org/etudescaribeennes/18967>

6.	Raymondville M, Rodriguez CA, Richterman A, Jerome G, Katz A, Gilbert H, et al. Barriers and facilitators influencing facility-based childbirth in rural Haiti: a mixed method study with a convergent design. BMJ Glob Health. 2020;5(8).

7.	International Centre for Missing & Exploited Children (ICMEC): Haiti- national child protection legislation. <https://www.icmec.org/wp-content/uploads/2018/08/ICMEC-Haiti-National-Legislation.pdf> (2018). Accessed 2024 11 April.
